# Serum expression signature of TUG1, MALAT1, miR-483, and miR-141 and their targets TGF-β1 and STAT3 in severe male factor infertility

**DOI:** 10.1038/s41598-025-03231-0

**Published:** 2025-05-27

**Authors:** Mahmoud A. Senousy, Olfat G. Shaker, Ahmed Gamal, Nesreen M. Aboraia, Ghada Ayeldeen

**Affiliations:** 1 Biochemistry Department, Faculty of Pharmacy, Cairo University, Kasr Al Ainy st., Cairo, 11562 Egypt; 2 Medical Biochemistry and Molecular Biology Department, Faculty of Medicine, Cairo University, Cairo, Egypt; 3 Andrology, Sexology, and STIs Department, Faculty of Medicine, Cairo University, Cairo, Egypt; 4 Dermatology and STDs Department, Faculty of Medicine, Fayoum University, Fayoum, Egypt

**Keywords:** Azoospermia, Infertility, LncRNA, MicroRNA, Oligozoospermia, Biochemistry, Molecular biology, Biomarkers, Diseases, Endocrinology, Medical research, Urology

## Abstract

Long non-coding RNAs and microRNAs have evolved as biomarkers and specific therapeutic targets for male infertility. We investigated the seminal plasma and serum expression profiles of TUG1, MALAT1, miR-483, and miR-141 and their targets TGF-β1 and STAT3 in severe male factor infertility. The potential of their serum levels as minimally invasive, clinically accessible, and more feasible biomarkers and their clinical correlations were explored. Thirty non-obstructive azoospermia (NOA) patients, 30 severe oligozoospermia (SO) patients, and 30 healthy fertile men (controls) were enrolled. Compared to controls, seminal plasma and serum TUG1 and miR-141 were downregulated; meanwhile, miR-483 and STAT3 were upregulated in both NOA and SO patients, while MALAT1 was upregulated in NOA but downregulated in SO patients. Seminal plasma TGF-β1 was higher in NOA patients than in controls and SO patients, while its serum levels were not statistically different between the studied groups. NOA patients showcased higher serum STAT3 mRNA expression than SO patients. Seminal plasma and serum levels of most of the tested markers were correlated among NOA and SO patients. Multivariate logistic analysis unraveled the association of serum TUG1 and miR-141 with NOA risk and serum TUG1 and miR-483 with SO risk. A serum-based panel of TUG1/miR-141 and TUG1/miR-483 discriminated NOA (AUC = 0.93) and SO (AUC = 0.972) from controls, respectively. Serum miR-141 was correlated with the hormonal profile in NOA patients. In SO patients, serum TUG1 was correlated with total testosterone levels and abnormal sperm motility, while serum MALAT1 was inversely correlated with the testicular volume. Conclusively, this study introduces a novel serum-based prediction panel of TUG1/miR-141 and TUG1/miR-483 to help enhance the accuracy of NOA and SO diagnosis. Serum MALAT1 and STAT3 could be useful in stratifying NOA and SO. These findings warrant more future investigations for their possible implication in male infertility diagnostics.

**Supplementary Information:**

The online version contains supplementary material available at 10.1038/s41598-025-03231-0.

## Introduction

Infertility is a major health problem across the world, affecting approximately 17.5% of adults globally^1,2^, with male factor infertility contributing to about 50% of these cases^2^. About 50% of couples have male factor infertility as a primary or contributing cause, and 20% have it as a single component^3^. The prevalence of male infertility rises globally, leading to multiple negative effects on the affected couple^3^. The age-standardized prevalence rate for male infertility in 2019 was estimated to be 1,402.98 per 100,000 population, with the highest prevalence occurring in the 30–34 years age group worldwide^4^.

Male infertility is brought on by aberrant sperm parameters in the male partner and is caused by congenital, acquired, or idiopathic factors that impair spermatogenesis or affect the sperm quality^3,5^. About 30–45% of idiopathic male infertility cases are accompanied by either severe oligozoospermia or azoospermia, which are characterized by very few or no spermatozoa in an ejaculate, respectively, thus requiring assisted reproductive technology^6,7^. In particular, azoospermia is the most severe manifestation of testicular failure, accounting for 15% of infertile men^3,8^.

In clinical settings, semen analysis is the standard procedure used to assess male infertility. However, it has several limitations and cannot always provide a reliable diagnosis, as semen irregularities do not necessarily rule out the possibility of normal fertility^9^. Furthermore, infertile men are routinely subjected to detailed medical history, physical examinations, hormonal and genetic assessments, and occasionally testicular biopsies or aspirations for precise diagnosis^10^. Despite these efforts, many of these approaches do not clarify the reasons behind male infertility and possess various drawbacks, such as being costly and labor-intensive (especially genetic and hormonal tests), difficult, and invasive (biopsy). Moreover, in many cases a testicular biopsy, hormonal profiling (e.g., FSH and inhibin B), and imaging are performed to differentiate the types of azoospermia, whether obstructive (caused by a blockage) or non-obstructive (caused by primary testicular failure and germ cell loss)^11^. On the other hand, the current hormonal therapies (e.g., exogenous FSH and hCG) have rather low efficacy in idiopathic male infertility, with low pregnancy/live-birth rates in controlled trials^12,13^. These limitations warrant the identification of new minimally invasive biomarkers to help assess the degree of residual spermatogenesis, detect sperm defects, distinguish the divergent causes of infertility, and/or diagnose subfertility in men.

To address this gap, incorporating newer approaches such as sperm DNA fragmentation testing and epigenetic assessments may improve prediction, diagnosis, and personalized management of male factor infertility^14,15^. A novel approach has suggested serum analysis of epigenetic markers in male infertile men as a promising diagnostic tool^16^.

Epigenetic alterations have evolved as key players in male fertility^11,15,17^. DNA methylation and non-coding RNAs (ncRNAs) are among the critical regulatory mechanisms impeccable for normal spermatogenesis^11,15,17^. Interestingly, compelling evidence highlighted the role of ncRNAs in defining sperm characteristics, including sperm morphology, motility, and count, and their entanglement in spermatogenesis, sperm maturation, and male infertility^18–22^. Indeed, multiple microRNAs (miRNAs) have been connected to azoospermia, oligozoospermia, asthenozoospermia (abnormal sperm motility), and teratozoospermia (abnormal sperm morphology)^20^. New sequencing and microarray techniques have identified an increasing number of spermatogenesis-associated long ncRNAs (lncRNAs) that are deregulated in male infertility^18,19^, but their precise functions and crosstalk with miRNAs/target mRNAs are undefined. Therefore, further investigations on such epigenetic factors are essential to develop therapeutic strategies to treat male infertility.

Taurine upregulated gene 1 (TUG1) is a highly conserved lncRNA and is functionally linked to male fertility. The loss of the Tug1 locus on chromosome 11 led to complete sterility in male mice due to severely impaired spermatogenesis^23^. Moreover, TUG1 has an important role in blood-testis barrier integrity of Sertoli cells (SC)^24^. Another spermatogenesis-associated lncRNA is metastasis-associated lung adenocarcinoma transcript 1 (MALAT1). It participates in sperm development and is enriched in the sperm nucleus and centralizes to the nuclear regions in the testicular interstitial cells^18,25^. The competing endogenous RNA mechanism of lncRNAs is highly involved in spermatogenesis through sponging miRNAs^19^. Of interest, miR-483-3p and miR-141-3p are direct targets of MALAT1^26–29^, and miR-141-3p is also a target of TUG1^30^. Intriguingly, miR-483-3p and miR-141-3p are among the detected miRNAs in seminal plasma^31^. miR-483-3p acts as a tumor suppressor in testicular seminoma via targeting matrix metalloproteinase 9^32^. Meanwhile, miR-141 is a potential regulator of spermatid differentiation^33^. Interestingly, both miRNAs regulate the transforming growth factor (TGF)-β and Janus kinase 2 (JAK2)/signal transducer and activator of transcription 3 (STAT3) signaling pathways^34–39^, impeccable regulators of spermatogenesis^40,41^. Indeed, TGF-β1 regulates reproductive tissue development and characteristic cyclic modification as characterized in Tgfb1 null mutant mice^40^. Meanwhile, the JAK2/STAT3 pathway is a crucial regulatory pathway of spermatogenesis and SC function^41^. Given their role in spermatogenesis, the clinical significance and predictive abilities of TUG1, MALAT1, miR-483, miR-141, and their crosstalk in infertile men are yet to be determined.

While ncRNAs have been profiled in sperm, seminal plasma, and/or testicular tissue of infertile men^21,22,31,33^, their serum expression and predictive potential remain underexplored. Clinically, serum biomarkers have the advantages of combining minimal invasiveness with lower variability, easier sampling in routine visits, easier standardization, robust reference ranges, and seamless integration into routine endocrine testing. Conversely, seminal plasma and testicular markers are limited by collection challenges, matrix complexity, pre-analytical variability, few validation assays, and little potential for high-throughput^42^, giving serum markers stronger prospects for routine clinical deployment.

In this context, this study aimed to investigate the seminal plasma and serum expression of TUG1, MALAT1, miR-483, and miR-141 and their networks TGF-β1 and STAT3 in infertile men with non-obstructive azoospermia (NOA) and severe oligozoospermia (SO). We then examined the correlation between their serum and seminal plasma profiles. The predictive and diagnostic potential of serum markers for both diseases and their correlations with hormonal profiles and semen quality parameters were explored.

## Subjects and methods

### Subjects

This case-control cross-sectional study included an overall 90 adult men, aged 18–60 years, allocated into 3 groups: group I: 30 healthy, fertile men served as controls, and two groups represented male factor infertility: group II: 30 infertile men with SO and group III: 30 infertile men with NOA. Subjects were referring to the Andrology, Sexology, and STIs Department, Kasr Al-Ainy Hospital, Faculty of Medicine, Cairo University, Cairo, Egypt, and Dermatology and STDs Department, Faculty of Medicine, Fayoum University, Fayoum, Egypt in the period from January to June 2024.

According to the WHO (2021), fertile men were defined as men whose partner had a natural conception with a confirmed time to pregnancy of < 12 months^43^. The female partners of infertile men did not use contraceptives and were not pregnant for one year. After referral to a gynecologist, a thorough full history and physical examination revealed no discernable cause for infertility.

After an informed consent, all subjects were evaluated using standard medical and surgical history taking, clinical and physical examination, semen analysis, hormonal assessment, and genetic analysis of sex chromosome abnormalities, along with ultrasound evaluation. Semen samples from all participants were collected after 3 days of not having sex. A computer-assisted semen analysis (Autosperm, Fertipro, Beernem, Belgium) was applied on liquefied semen samples. The sperm morphology was then assessed using a phase-contrast microscope and sperm Mac stain (Fertipro, Belgium) according to the WHO criteria (2010)^7^. NOA was defined as a complete absence of sperm in an ejaculate in three different semen analyses at least two weeks apart and centrifugation of semen specimens for 15 min at 3000*g* at room temperature with high-powered microscopic examination of the pellet. SO is defined as very low sperm count with < 5 million sperm/mL of an ejaculate^6,7^. Testicular size was considered normal if ≥ 12 mL, moderate when 6–12 mL, and small if < 6 mL.

In accordance with WHO standards (2021), the control group comprised age-matched, healthy, and fertile men whose fertility was confirmed by semen analysis and documented pregnancy/live-birth of their partners. They had fathered at least one child without the use of assisted reproductive techniques over the same time period as the cases.

The criteria for inclusion were men aged between 18 and 60 having no evidence of inflammatory granuloma or malignancy. Strict exclusion criteria were taken into consideration. These comprised individuals with recognized medical causes of infertility, such as chromosomal abnormalities, Y chromosome microdeletions, mumps, varicocele, retrograde ejaculation, pathologies of the vas deferens or epididymis, and cryptorchidism. Furthermore, patients having obstructive azoospermia, teratozoospermia, mild and moderate oligozoospermia according to spermogram, or endocrine disorders, those on hormonal medications, alcohol consumption, or drug intake, or with leukocytospermia were excluded. Moreover, men with known comorbidities, including cancer, diabetes, autoimmunity, gastrointestinal disease, and kidney or lung disorders, were excluded.

All activities involving human subjects were carried out in full compliance with government policies and the Helsinki Declarations. All patients and controls have received and signed a formal informed consent agreement. The study protocol and experimentations were approved by the Scientific Research Ethics Committee, Fayoum University, Fayoum, Egypt (Approval number R559).

### Seminal plasma and serum separation and analysis of serum hormonal profile

At admission, semen samples were collected form all participants with the standard protocol. The seminal plasma was separated during the sperm selection method (centrifugation) and stored at – 80 °C for any further assay. Additionally, whole blood samples (approximately 5 mL) were collected from each subject by vein puncture into yellow-gel vacutainer tubes. After 30 min, the serum was obtained by centrifugation at 4 °C for 10 min at 4000 rpm. Until analysis, aliquots of the serum were frozen at – 80 °C. Serum concentrations of total testosterone (TTST), luteinizing hormone (LH), follicle-stimulating hormone (FSH), prolactin (PRL), and estradiol (E2) were determined using commercially available ELISA kits.

### Bioinformatics analysis for LncRNA target prediction

The Human MicroRNA Disease Database HMDD version 4.0 (http://www.cuilab.cn/hmdd) and PubMed were used to detect the miRNAs related to azoospermia. Then, the ENCORI/starBase database (https://rnasysu.com/encori/) was queried to identify predicted miRNA-lncRNA interactions. Candidate miRNA-lncRNA interactions were subsequently cross-validated using experimentally validated datasets available in starBase v3.0. miRNA target predictions were further established using several large databases, including starBase, miRWalk (http://mirwalk.umm.uni-heidelberg.de/), which primarily rely on computational predictions, and miRPathDB 2.0 (https://mpd.bioinf.uni-sb.de/), which incorporates both computationally predicted and experimentally validated target interactions. Further experimental validation of miRNA-lncRNA and miRNA-target gene interactions was confirmed using PubMed search. Construction of a molecular interactions network was done using the Pathway Studio online tool (https://mammal-profservices.pathwaystudio.com/app/search).

### Gene expression assays

The miRNeasy RNA extraction kit (Qiagen, Valencia, CA, USA) was employed for extricating the total RNA from 200 µL of seminal plasma or hemolysis-free serum following the manufacturer’s protocol. The RNA concentration and purity were determined using the Nano Drop2000 (Thermo Scientific, Waltham, MA, USA). Following the manufacturers’ recommendations, cDNA was synthesized using the RevertAid First Strand cDNA Synthesis kit (Thermo Fisher Scientific, USA) for lncRNAs and the miRCURY LNA RT kit (Qiagen, Valencia, USA) for miRNAs. For quantitative PCR, the cDNA samples were then amplified using the miRCURY LNA SYBR Green PCR kit and customized primers (eurofins Genomics, GmbH, Germany) according to the manufacturer’s instructions. Primers were predesigned using the primer3web software version 4.1.0 (https://primer3.ut.ee/), and their target specificity was examined using the NCBI primer-BLAST tool. The primers’ sequences are listed in Table 1. GAPDH and SNORD68 were employed as internal controls for normalizing lncRNAs and miRNAs, respectively, as documented in earlier studies^44,45^. To assure the specificity of the corresponding PCR products and the absence of hairpin or primer dimers, a melting curve analysis was performed. Fold change was calculated using the 2^−ΔΔCt^ formula for relative quantification.

**Table 1:** The primer sequences used in the study.

Gene	Primer sequence (5′–3′)
TUG1	**F**: TAGCAGTTCCCCAATCCTTG
	**R**: CACAAATTCCCATCATTCC
MALAT1	F: AAAGCAAGGTCTCCCCACAAG
	R: GGTCTGTGCTAGATCAAAAGGCA
STAT3	F: TCTGCCGGAGAAACAGTTGG
	R: AGGTACCGTGTGTCAAGCTG
miR-483-3p	F: ATCACTCCTCTCCTCCCGTC
miR-141-3p	F: CAUCUUCCAGUACAGUGUUGGA
SNORD68	F: CGCGTGATGACATTCTCC
GAPDH	F: GAAGGTCGGAGTCAACGGATT
	R: CGCTCCTGGAAGATGGTGAT

*MALAT1* metastasis-associated lung adenocarcinoma transcript 1, *STAT3* signal transducer and activator of transcription 3, *TUG1* taurine upregulated gene 1.

### Assay of serum TGF-β1

Seminal plasma and serum TGF-β1 protein levels were analyzed using Human TGF-beta 1 ELISA kit (Invitrogen). The results are given in ng/mL.

### Sample size analysis

The sample size was calculated using the G power software 3.1.9.7 based on a large effect size, as described in a previous study^22^. Using the F test of ANOVA, effect size f = 0.4, alpha error of 0.05, and a power of 0.8 (two-tailed), the minimum total sample size was calculated to be 66 participants for 3 independent groups (22 per group). To accommodate for possible dropouts, we increased the sample size to 90 participants (power = 92.6%).

### Statistical analysis

Statistical analysis was computed utilizing the GraphPad Prism version 7.0 (GraphPad Software, CA, USA). Prior to analysis, missing data were assessed. Cases with missing values were minimal and handled using mean/median imputation. Quantitative variables were summed with the use of mean and standard deviation or median (25–75% percentiles). Categorical variables were expressed as numbers and percentages. The D’Agostino & Pearson and Shapiro-Wilk tests were used to assess the normal distribution of parameters. Accordingly, the t-test or one-way analysis of variance test followed by Tukey’s post-hoc test was used to compare parametric data, while non-parametric data were compared using the Mann–Whitney U test or Kruskal–Wallis followed by Dunn’s multiple comparison test when appropriate. The Chi-square test was applied to compare the categorical variables. Correlations between variables were computed using the Spearman rank correlation coefficient. The association of variables with the risk of developing the disease was tested using logistic regression analyses. We gathered the statistically significant parameters from the univariate logistic analysis into a stepwise forward multivariate analysis, adjusted for confounders, and applied cross-validation. Then, receiver-operating characteristic (ROC) curves were constructed from the logistic regression models for diagnostic potential assessment, and the area under the curve (AUC) was calculated. AUC > 0.7 or > 0.9 was set as a criterion to designate the variable as a promising or an excellent biomarker, respectively. Cutoff values were selected using the Youden index (sensitivity + specificity – 1) to optimize sensitivity and specificity. The final predictors configured from the adjusted multivariate analysis were utilized to construct a predictive model. Statistical significance was assumed when the two-tailed *P* value was < 0.05.

In addition to *P* values, effect sizes were calculated to assess the magnitude of differences in serum hormone levels between the three studied groups. Eta squared (η^2^) or epsilon-squared (ε^2^) values were used for three-group comparisons for parametric (ANOVA test) or non-parametric (Kruskal–Wallis test) data, respectively, with values η^2^ or ε^2^ ≈ 0.01 considered small, ≈ 0.06 medium, and 0.14 or higher considered large effect size.

## Results

### Demographic, clinical, and laboratory data of the studied groups

#### Demographic and hormonal profile of SO and NOA patients

As shown in Table 2, there were no significant differences in smoking status, serum TTST, PRL, and E2 levels between the three studied groups (fertile controls, SO, and NOA). The corresponding effect sizes (Eta-squared, η^2^) for hormonal data were: TTST η^2^ = 0.021 (small), PRL η^2^ = 0.03 (small), and E2 η^2^ = 0.003 (small, very negligible).

**Table 2:** The demographic, clinical, and laboratory data of the studied groups.

Parameter	Healthy controls (*n* = 30)	SO (*n* = 30)	NOA (*n* = 30)	*P* value
Age (years), n (%)	31.37 ± 8.54	30.40 ± 9.11	32.80 ± 6.82	0.33
Range (years)	20–55	18–60	21–44	
Smoking, n (%)	12 (40)	15 (50)	18 (60)	0.3
TTST (ng/mL)	4.75 ± 2.09	5.40 ± 2.18	4.64 ± 2.69	0.4
PRL (ng/mL)	10.62 ± 4.74	12.86 ± 6.66	12.17 ± 4.73	0.24
E2 (pg/mL)	29.58 ± 7.50	29.74 ± 12.95	30.89 ± 10.86	0.88
LH (IU/L)	4.98 ± 1.84	6.86 ± 3.40	10.13 ± 5.27^#$^	**< 0.0001**
FSH (IU/L)	3.5 (2.58–4.6)	6.95 (3.6–10.2)^#^	8.8 (5.97–24.05)^#^	**< 0.0001**
Right testicular volume	13.57 ± 2.83	11.58 ± 3.26	7.35 ± 4.32^#$^	**< 0.0001**
Left testicular volume	12.50 ± 3.18	10.54 ± 3.65	7.48 ± 4.35^#$^	**< 0.0001**
Testicular volume (mL), n (%)				**< 0.0001**
Normal (≥ 12)	19 (63.33)	13 (43.33)	6 (20)	
Moderate (6–12)	11 (36.67)	16 (53.34)	13 (43.33)	
Small (< 6)	0 (0)	1 (3.33)	11 (36.67)	
Semen volume (mL)	2.56 ± 1.28	2.09 ± 1.26	2.25 ± 1.44	0.13
Sperm count (million/mL)	45.5 (33.5–62.03)	3.2 (1.2–4.3)	–	**< 0.0001**
Total motility (%)	61.0 ± 6.87	30.0 ± 15.10	–	**< 0.0001**
Non-progressive motility (%)	38.33 ± 5.47	22.17 ± 14.42	–	**< 0.0001**
Abnormal forms (%)	55.17 ± 6.23	66.03 ± 9.95	–	**< 0.0001**

*E2* estradiol 2, *FSH* follicle-stimulating hormone, *LH* luteinizing hormone, *NOA* non-obstructive azoospermia, *PRL* prolactin, *SO* severe oligozoospermia, *TTST* total testosterone.

Data of the studied groups are presented as mean ± SD, median (25–75% percentiles), or number (percentage). Categorical data were compared using Chi-square test. Numerical data from 3 groups were compared using the one-way ANOVA test followed by Tukey’s post-hoc test except FSH was compared using the non-parametric Kruskal–Wallis test followed by Dunn’s multiple comparison test, while data from two groups were compared using the t test except the sperm count was compared using the non-parametric Mann–Whitney U test.

^#^Significant difference from healthy controls.

^$^Significant difference from SO. Statistical significance was set at *P* < 0.05 (bold).

NOA patients showed higher serum LH levels than the other two groups (*P* < 0.0001). The η^2^ for LH across the three groups was 0.248 (large effect size). Both NOA and SO patients exhibited significantly higher serum FSH levels than the fertile controls (*P* < 0.0001), while there were no comparable differences in FSH levels when these two groups were compared (*P* > 0.05). For FSH, the data were not normally distributed and a non-parametric effect size (epsilon-squared, ε^2^) was calculated, yielding ε^2^ = 0.798 (very large). To note, NOA patients showcased significantly smaller testicular size than the other two groups (*P* < 0.0001).

#### Results of semen analysis

Semen analysis revealed markedly distorted sperm quality in the SO patients. Despite having a comparable semen volume with the controls, they showcased a remarkable reduction in sperm count and motility by 92.7% and 50%, respectively, and an increase in abnormal forms by 20% compared to the healthy fertile controls (*P* < 0.0001).

### Results of the target prediction analysis

**Fig. 1:**
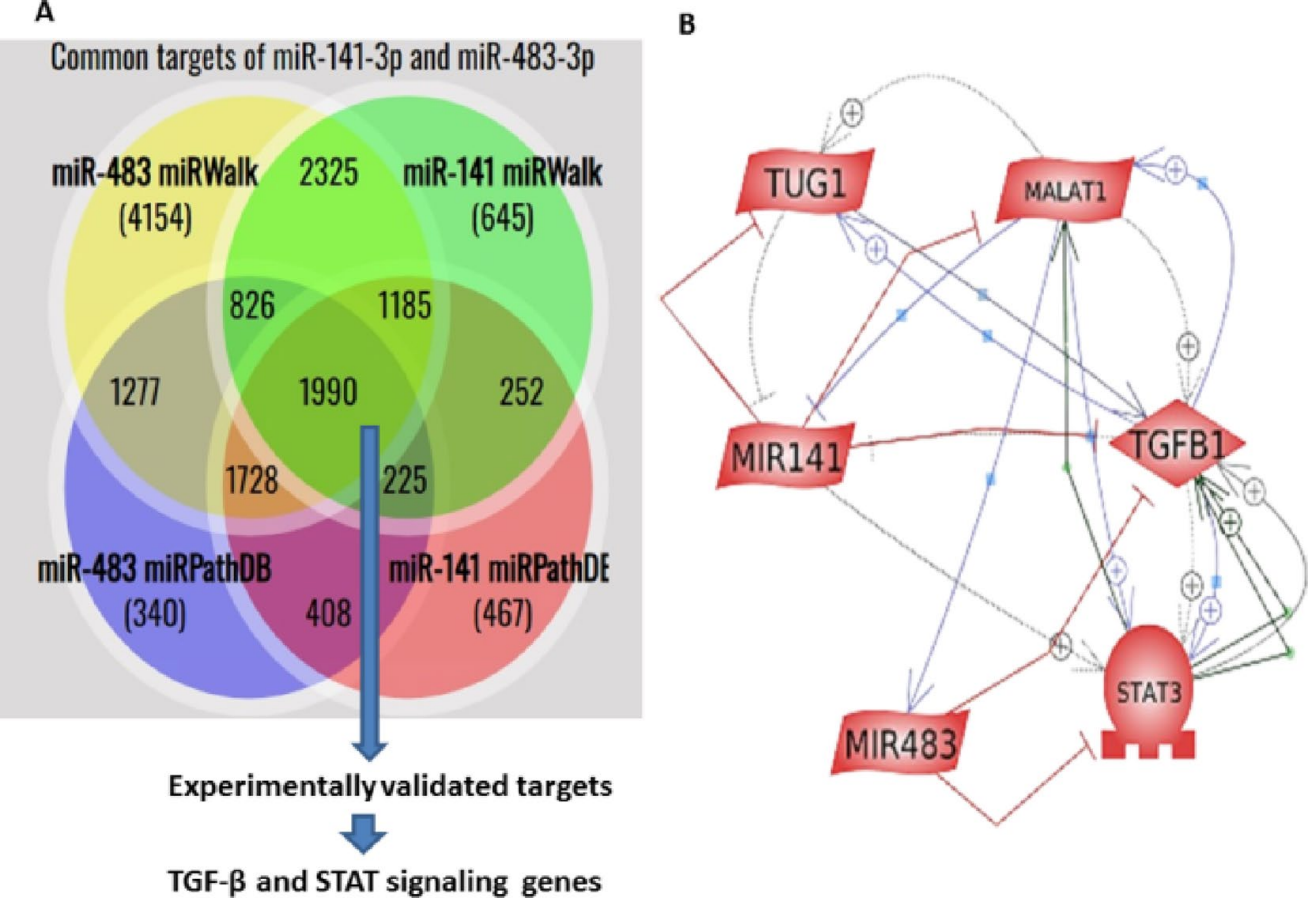
Target prediction and visualization of the lncRNA/miRNA/target genes interaction network. **A** Venn diagram to select the common predicted targets of miR-483-3p and miR-141-3p using miRPathDB 2.0 and miRWalk databeses. **B** Visualization of molecular interactions between the investigated parameters using the Pathway Studio online tool. MALAT1, metastasis-associated lung adenocarcinoma transcript 1; *STAT3* signal transducer and activator of transcription 3, *TGF-β1* transforming growth factor-β1, *TUG1* taurine upregulated gene 1.

The HMDD database and PubMed search revealed multiple miRNAs related to azoospermia with evidence of testicular tissue or seminal expression. Out of them, miR-141-3p and miR-483-3p are direct targets of MALAT1 as confirmed by the ENCORI/starBase database (TDMDScore = 0.97 and 1.2, respectively) and experimental validation in various models^26–29^. Also, there is evidence that miR-141-3p is an experimentally verified downstream target of TUG1^30^. Target prediction databases revealed common predicted targets between miR-483-3p (MIMAT0002173) and miR-141-3p (MIMAT0000432) (Fig. 1A). We filtered the output to experimentally validated targets. In particular, TGF-β and STAT signaling-related genes, including TGFB1, TGFB2, TGFBR1, TGFBR2, STAT1, STAT3, STAT4, STAT5A, and STAT5B were revealed, indicating that these two miRNAs intersect at the TGF-β and STAT signaling pathways. Herein, we focused on TGF-β1 and STAT3 as downstream targets, as their interaction with miR-483 and miR-141 was experimentally validated in cell lines^34,35,37,46^. Figure 1B portrays a network of mutual interactions of TUG1, MALAT1, miR-483, miR-141, TGF-β1, and STAT3 visualized using the Pathway Studio.

### Dysregulated expression of seminal plasma TUG1, MALAT1, miR-483, and miR-141 and their targets TGF-β1 and STAT3 in SO and NOA patients

#### Seminal plasma TUG1, MALAT1, miR-483, and miR-141 expression in SO and NOA patients

**Fig. 2:**
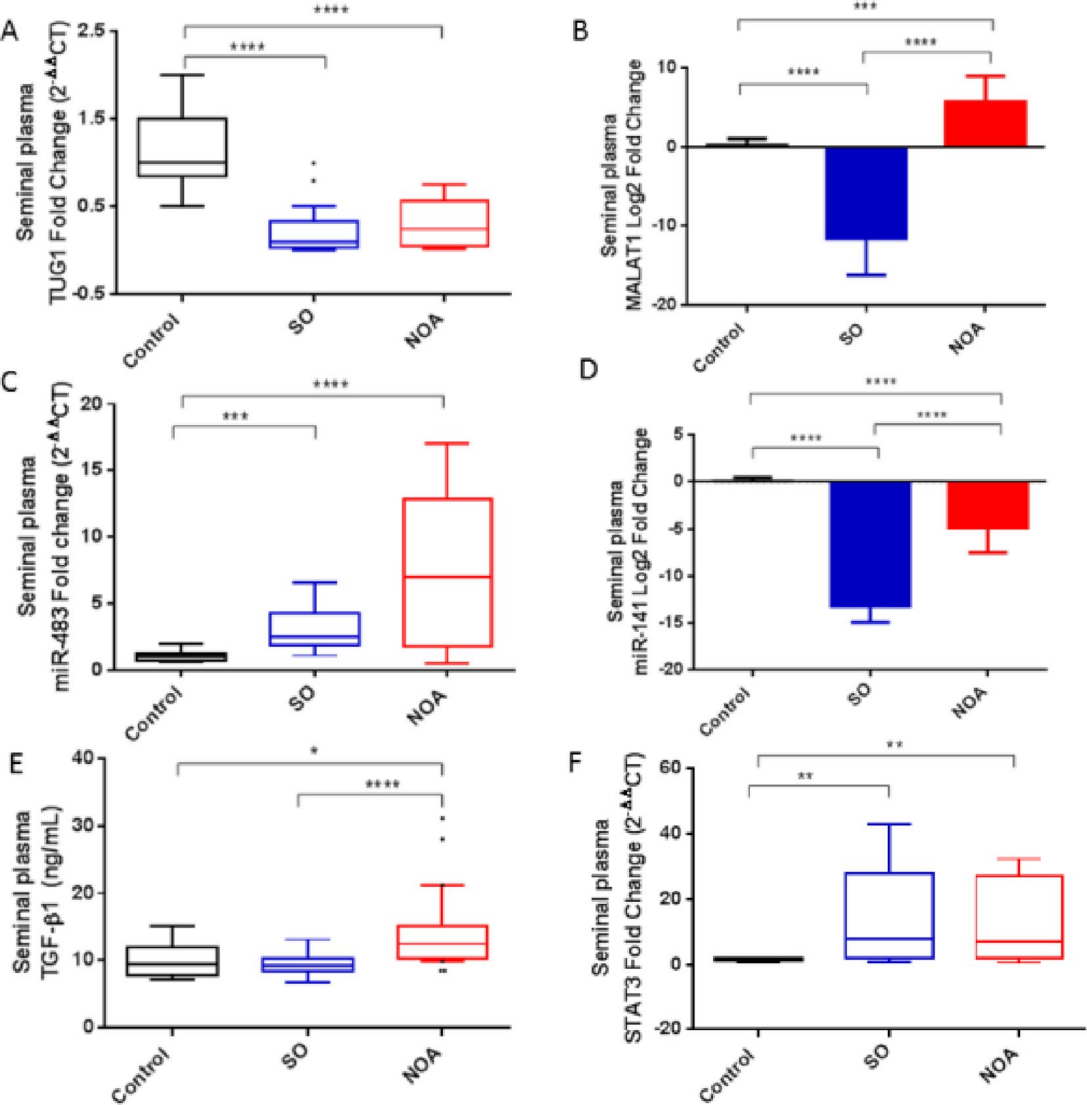
Seminal plasma expression levels of the investigated parameters in the studied groups. NOA, *n* = 30, SO, *n* = 30, healthy fertile controls, *n* = 30. Data are expressed as box blot (Tukey) or mean ± SD. Data were compared using Kruskal–Wallis test followed by Dunn’s multiple comparison test. Data of MALAT1 and miR-141 were log2 transformed for clearer presentation and compared using parametric tests, one-way ANOVA followed by Tukey. *P < 0.05, **P < 0.01, ***P < 0.001, ****P < 0.0001.

TUG1 expression was downregulated in the seminal plasma of both SO and NOA patients compared to controls, with comparable levels in both infertile groups (Fig. 2A). On the other hand, upregulated MALAT1 expression was observed in the seminal plasma of NOA patients, whereas SO patients showed markedly downregulated MALAT1 expression levels compared to controls (Fig. 2B).

Compered to controls, the expression of miR-483 in the seminal plasma was upregulated (Fig. 2C), whereas miR-141 was downregulated (Fig. 2D) in both the SO and NOA groups. Seminal plasma miR-141 levels were significantly lower in NOA than those in the SO patients, while miR-483 were not significantly different between the two groups.

#### Seminal plasma TGF-β1 and STAT3 expression in SO and NOA patients

Seminal plasma TGF-β1 protein levels were higher in NOA patients than in controls and SO patients (Fig. 2E), while they were comparable in SO patients and fertile controls. Meanwhile, seminal plasma STAT3 mRNA expression was upregulated in both SO and NOA patients compared to controls, with no significant difference between the two groups (Fig. 2F).

### Dysregulated expression of serum TUG1, MALAT1, miR-483, and miR-141 and their target STAT3 in SO and NOA patients

#### Serum TUG1, MALAT1, miR-483, and miR-141 expression in SO and NOA patients

**Fig. 3:**
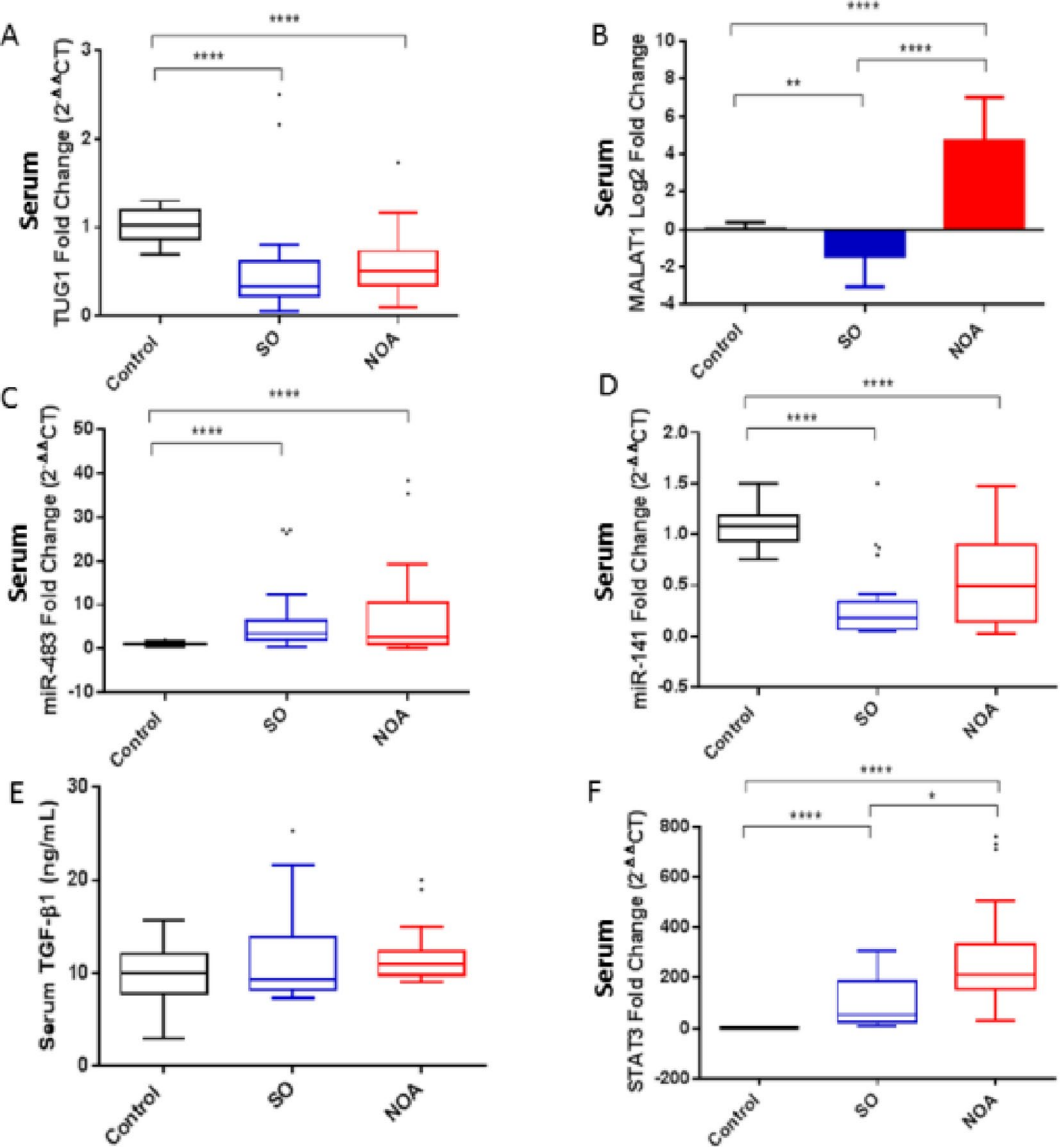
Serum expression levels of the investigated parameters in the studied groups. NOA, *n* = 30, SO, *n* = 30, healthy fertile controls, *n* = 30. Data are expressed as box blot (Tukey) or mean ± SD. Data were compared using Kruskal–Wallis test followed by Dunn’s multiple comparison test. Data of MALAT1 were log2 transformed for clearer presentation and compared using parametric tests, one-way ANOVA followed by Tukey. *P < 0.05, **P < 0.01, ****P < 0.0001.

A substantial downregulation of serum TUG1 expression was observed in SO and NOA patients compared to controls, with comparable levels in both infertile groups (Fig. 3A). On the other hand, NOA patients showcased highly upregulated levels of serum MALAT1 expression, whereas SO patients showed significantly downregulated MALAT1 levels compared to controls (Fig. 3B).

Regarding miRNAs, serum miR-483 was upregulated (Fig. 3C), while serum miR-141 was downregulated (Fig. 3D) in both SO and NOA groups compared to controls. Levels of both miRNAs were not significantly different when SO and NOA groups were compared.

#### Serum TGF-β1 and STAT3 expression in SO and NOA patients

Noteworthy, there was no statistical difference in serum TGF-β1 protein levels between the three studied groups (Fig. 3E). Meanwhile, serum STAT3 mRNA expression was markedly augmented in both SO and NOA patients compared to the control group, with significantly higher levels in NOA patients than those in SO patients (Fig. 3F). The median (25–75% percentiles) of the fold change of RNA expression in SO and NOA groups is presented in Table 3.

**Table 3:** Expression levels of serum TUG1, MALAT1, miR-483, and miR-141, and STAT3 in SO and NOA patients compared to healthy controls.

Parameter	SO (*n* = 30)	NOA (*n* = 30)	*P* value^#^	Post hoc pairwise comparison *P* value^$^
	Fold change comparing to healthy controls			
TUG1	0.33 (0.22–0.61)	0.51 (0.35–0.73)	**< 0.0001**	^a^ **< 0.0001**
				^b^ **< 0.0001**
				^c^ **ns**
MALAT1	0.365 (0.23–0.51)	25.1 (13.82–92.85)	**< 0.0001**	^a^ **< 0.01**
				^b^ **< 0.0001**
				^c^ < **0.0001**
miR-483	3.45 (2.03–6.36)	2.69 (0.99–10.36)	**< 0.0001**	^a^ **< 0.0001**
				^b^ **< 0.0001**
				^c^ ns
miR-141	0.18 (0.07–0.33)	0.49 (0.14–0.90)	**< 0.0001**	^a^ **< 0.0001**
				^b ^ **< 0.0001**
				^c^ **<** ns
STAT3	53.67 (22.63–188.8)	212.3 (154.8–332.4)	**< 0.0001**	^a^ **< 0.0001**
				^b^ **< 0.0001**
				^c^ **< 0.05**

The median (25–75% percentiles) are used to present the data.

*ns* non-significant (*P* > 0.05), *MALAT1* metastasis-associated lung adenocarcinoma transcript 1, *STAT3* signal transducer and activator of transcription 3, *TUG1* taurine upregulated gene 1.

^#^Kruskal–Wallis test.

^$^Dunn’s multiple comparisons test.

^a^SO versus control.

^b^NOA versus control.

^c^NOA versus SO. Statistical significance was set at *P* < 0.05 (bold).

### Correlation between seminal plasma and serum levels of the investigated markers in NOA and SO patients

**Fig. 4:**
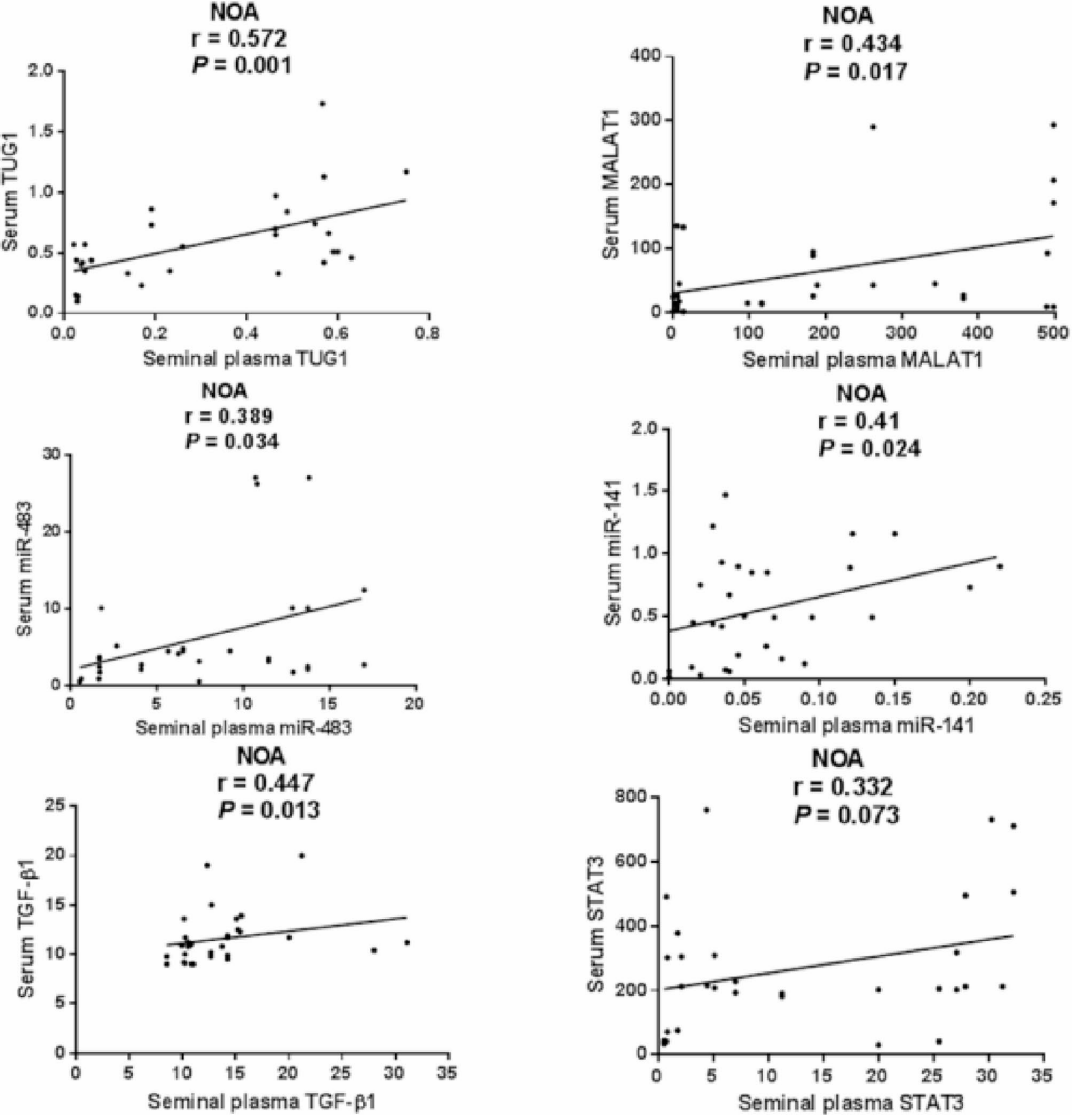
Correlation between seminal plasma and serum expression levels of the investigated parameters among NOA patients. Data were analyzed using Spearman correlation, *n* = 30. Statistical significance was set at *P* < 0.05.

As depicted in Fig. 4, seminal plasma and serum levels of TUG1 (Spearman *r* = 0.572, *P* = 0.001), MALAT1 (*r* = 0.434, *P* = 0.017), miR-483 (*r* = 0.389, *P* = 0.034), miR-141 (*r* = 0.410, *P* = 0.024), and TGF-β1 (*r* = 0.447, *P* = 0.013) were significantly correlated among NOA patients; however, STAT3 expression levels were not significantly correlated (*r* = 0.332, *P* = 0.073) .

**Fig. 5:**
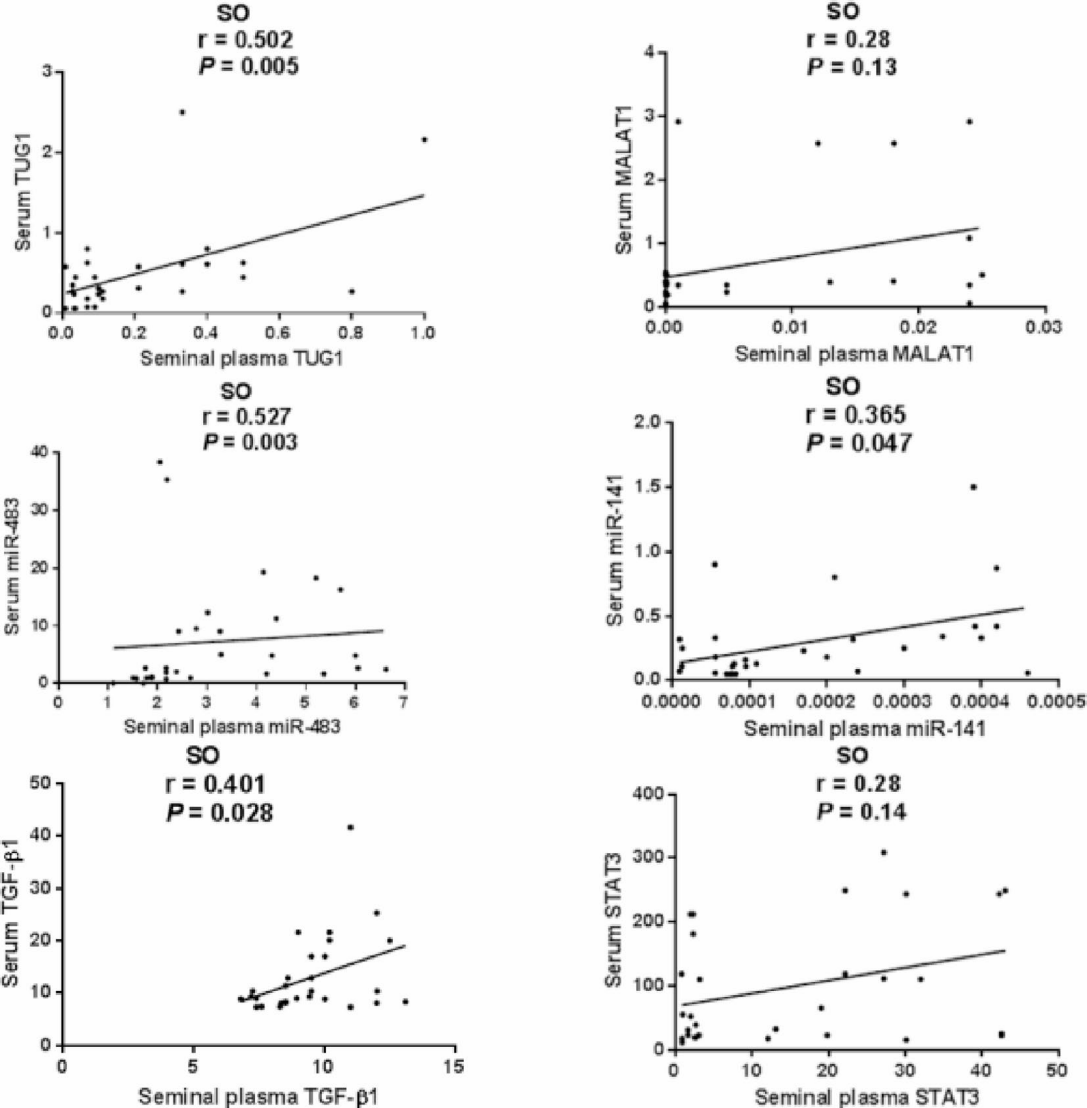
Correlation between seminal plasma and serum expression levels of the investigated parameters among SO patients. Data were analyzed using Spearman correlation, *n* = 30. Statistical significance was set at *P* < 0.05.

Among SO patients, seminal plasma and serum levels of TUG1 (Spearman *r* = 0.502, *P* = 0.005), miR-483 (*r* = 0.527, *P* = 0.003), miR-141 (*r* = 0.365, *P* = 0.047), and TGF-β1 (*r* = 0.401, *P* = 0.028) were significantly correlated; however, MALAT1 (*r* = 0.280, *P* = 0.13) and STAT3 (*r* = 0.280, *P* = 0.14) expression levels were not significantly correlated (Fig. 5).

### Serum TUG1 and miR-141 are associated with NOA, while TUG1 and miR-483 are associated with SO in the multivariate logistic analysis

Given the limitations of analyzing semen markers in clinical practice, we focused in this study on serum markers as less tedious, clinically accessible, and more feasible biomarkers. To figure out the predictor variables that might be connected to the probability of developing NOA or SO, binary logistic regression analyses were conducted between NOA vs. controls (Table 4) and SO vs. controls (Table 5) using serum levels of the tested markers.

**Table 4:** Association of molecular parameters with NOA using logistic regression analysis.

Parameter	Beta-coefficient	SE	*P* value	OR	95% CI
Univariate analysis					
TUG1	− 5.3232	1.3379	**0.0001**	0.0049	0.0004, 0.0671
MALAT1	0.9460	0.6373	0.1377	2.5753	0.7385, 8.9803
miR-483	1.2182	0.5269	**0.0208**	3.3813	1.2039, 9.4963
miR-141	− 5.6422	1.5255	**0.0002**	0.0035	0.0002, 0.0705
STAT3	1.4721	528.5658	0.9978	4.3582	–
Multivariate analysis^a^					
TUG1	− 3.3906	1.5210	**0.0258**	0.0337	0.0017, 0.6639
miR-483	0.6005	0.5464	0.2717	1.8230	0.6248, 5.3193
miR-141	− 5.6528	1.9444	**0.0036**	0.0035	0.0001, 0.1585
Constant	4.2079	3.2996			

Univariate logistic regression analyses were conducted using NOA cases, *n* = 30 and healthy controls, *n* = 30. Variables that showed statistical significance in the univariate analysis were then fed into a multivariate logistic analysis stepwise-forward model. *X*^2^ of the model = 54.45, *P* = 0.0000. Cross-validation was applied. Statistical significance was set at *P* < 0.05 (bold).

*CI* confidence interval, *OR* odds ratio.

^a^Controlled by smoking [OR (95%CI) = 4.5008 (0.5687–35.6186)] and age [OR (95%CI) = 1.0539 (0.9207–1.2062)] as confounders.

**Table 5:** Association of molecular parameters with SO using logistic regression analysis.

Parameter	Beta-coefficient	SE	*P* value	OR	95%CI
Univariate analysis					
TUG1	− 3.6268	0.9795	**0.0002**	0.0266	0.0039, 0.1814
MALAT1	− 1.1174	0.5228	**0.0326**	0.3271	0.1174, 0.9115
miR-483	2.2278	0.6679	**0.0009**	9.2793	2.5062, 34.3567
miR-141	− 7.2830	1.8515	**0.0001**	0.0007	0.0001, 0.0259
STAT3	4.1747	1459.2589	0.9977	65.0181	–
Multivariate analysis^a^					
TUG1	− 5.7169	1.5149	**0.0002**	0.0033	0.0002, 0.0641
MALAT1	− 0.4960	0.7005	0.4789	0.6089	0.1548, 2.4036
miR-483	1.1936	0.3316	**0.0003**	3.2988	1.7221, 6.3191
Constant	1.9096	2.0421			

Univariate logistic regression analyses were conducted using SO cases, *n* = 30 and healthy controls, *n* = 30. Variables that showed statistical significance in the univariate analysis were then fed into a multivariate logistic analysis stepwise-forward model. *X*^2^ of the model = 54.7, *P* = 0.0000. Cross-validation was applied. Statistical significance was set at *P* < 0.05 (bold).

*CI* confidence interval, *OR* odds ratio.

^a^Controlled by smoking [OR (95%CI) = 1.8601 (0.2352–14.7132)] and age [OR (95%CI) = 0.9961 (0.9100–1.0903)] as confounders.

The univariate analysis uncovered serum TUG1, miR-483, and miR-141 expression levels as significant predictors associated with the probability of developing NOA. Interestingly, the multivariate analysis, adjusted with smoking status and age, unraveled the association of serum TUG1 [adjusted OR (95%CI) = 0.0337 (0.0017–0.6639), *P* = 0.0258] and miR-141 [adjusted OR (95%CI) = 0.0035 (0.0001–0.1585), *P* = 0.0036] with NOA risk (Table 4).

For SO, serum TUG1, MALAT1, miR-483, and miR-141 expression levels were significant predictors associated with the risk of developing SO in the univariate analysis. Intriguingly, the multivariate analysis, adjusted with smoking status and age, uncovered the association of serum TUG1 [adjusted OR (95%CI) = 0.0033 (0.0002–0.0641), *P* = 0.0002] and miR-483 [adjusted OR (95%CI) = 3.2988 (1.7221–6.3191), *P* = 0.0003] with SO risk (Table 5).

### Single predictors showed discriminative potential using ROC curves constructed from the univariate logistic regression models

**Fig. 6:**
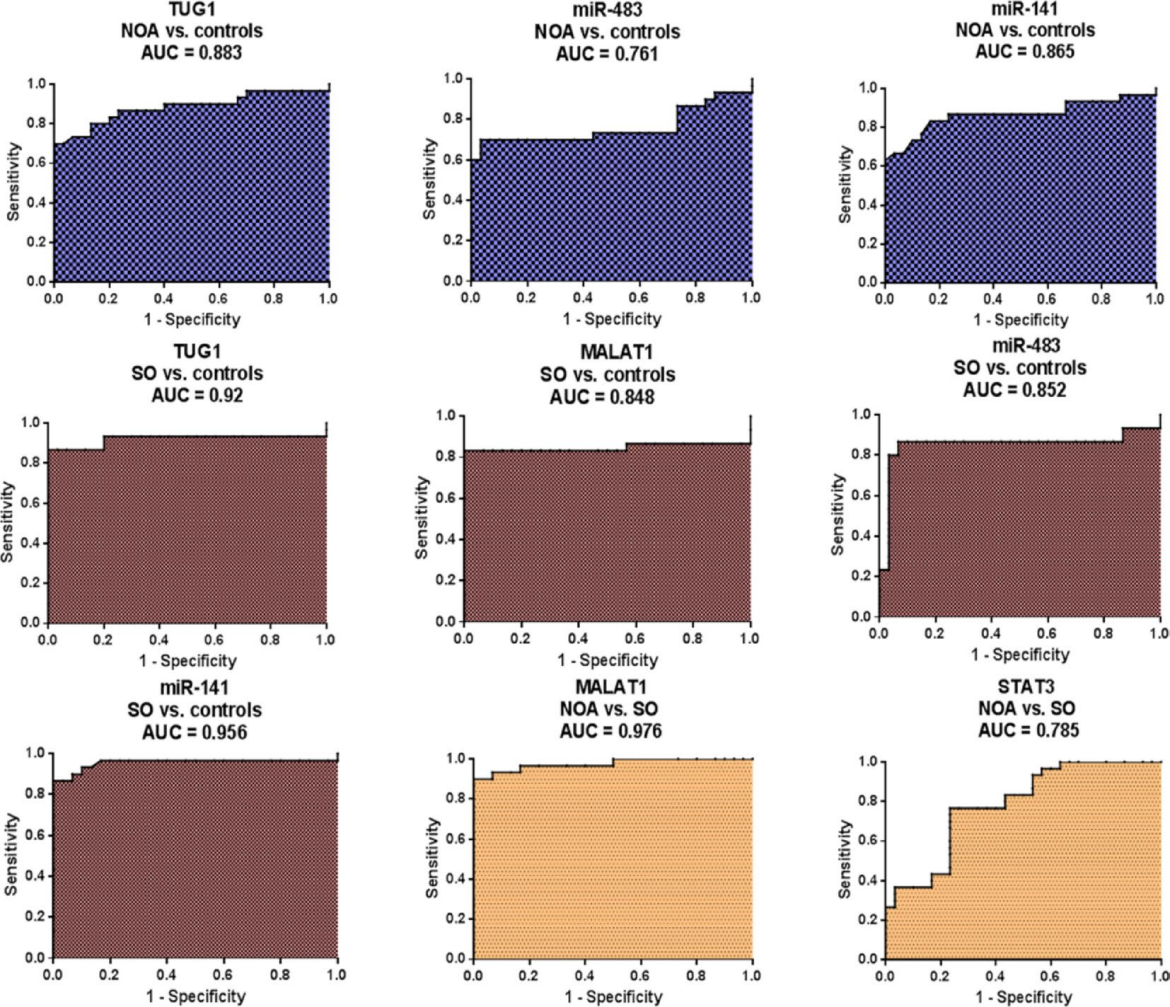
Diagnostic performance of serum expression levels of the single markers using ROC curve analysis. The analysis was performed to distinguish NOA (*n* = 30), SO (*n* = 30), and healthy controls (*n* = 30).

To configure the diagnostic potential of the serum level of each single variable associated with NOA or SO, ROC curves were constructed after binary classification in the univariate logistic regression models as portrayed in Fig. 6. The output of ROC analyses, including AUC (95%CI), *P* value, and sensitivity and specificity at the best cutoff values chosen using the Youden index on the ROC curve, is listed in Table 6.

**Table 6:** Diagnostic performance of the individual parameters.

Parameter	AUC	95%CI	*P* value	Cutoff (fold)	Sensitivity (%)	Specificity (%)
NOA versus healthy controls						
TUG1	0.883	0.7894, 0.9762	**< 0.0001**	< 0.67	70	100
miR-483	0.761	0.6238, 0.8973	**0.0005**	> 1.66	70	96.67
miR-141	0.865	0.7624, 0.9676	**< 0.0001**	< 0.9	83.33	83.33
SO versus healthy controls						
TUG1	0.92	0.8295, 1.011	**< 0.0001**	< 0.66	86.67	100
MALAT1	0.848	0.7231, 0.9725	**< 0.0001**	< 0.61	83	100
miR-483	0.852	0.7334, 0.9711	**< 0.0001**	> 1.67	86.67	96.67
miR-141	0.956	0.8904, 1.022	**< 0.0001**	> 0.58	90	100
NOA versus SO						
MALAT1	0.976	0.9395, 1.012	**< 0.0001**	> 2.6	93.33	93.33
STAT3	0.785	0.6712, 0.9000	**0.0002**	> 17.46	76.76	76.76

The value at which the sum of sensitivity and specificity is maximized was identified as the cutoff value using Youden index on the ROC curve. Cutoff values are expressed in fold change. NOA, *n* = 30; SO, *n* = 30; healthy controls, *n* = 30. CI, confidence interval. Statistical significance was set at *P* < 0.05 (bold).

ROC analysis displayed serum TUG1, miR-141, and miR-483 as biomarkers with promising diagnostic potential for NOA with AUCs (NOA vs. controls) = 0.883, 0.865, and 0.761, respectively. Noticeably, TUG1 and miR-141 were superior to miR-483.

In addition, serum TUG1 and miR-141 had excellent diagnostic potential (AUCs = 0.92 and 0.956, respectively), while serum miR-483 and MALAT1 were promising biomarkers (AUCs = 0.852 and 0.848, respectively) to distinguish SO patients from healthy fertile controls.

Analysis of significant variables between the NOA and SO groups revealed serum MALAT1 (AUC = 0.976) and STAT3 (AUC = 0.785) as excellent and promising biomarkers distinguishing the two cases, respectively.

### Enhancing the diagnostic accuracy of NOA and SO using combined serum-based predictive panels

Based on our molecular markers, the output of adjusted multivariate analysis (Tables 4 and 5) was employed to construct a serum-based predictive panel combining the final predictors: TUG1 + miR-141 for NOA and TUG1 + miR-483 for SO.

#### Construction of a predictive panel for NOA

**Fig. 7:**
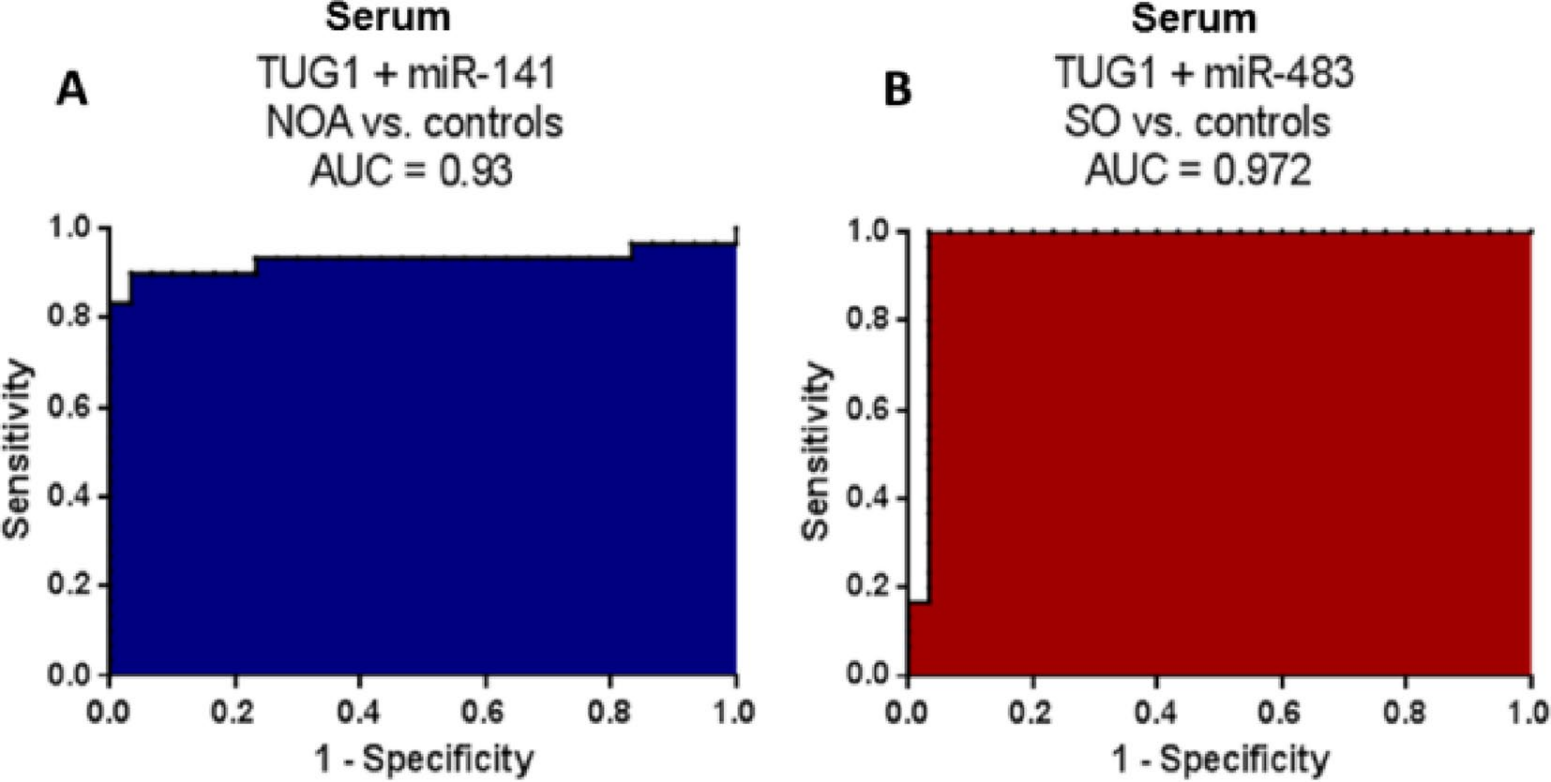
Diagnostic accuracy of serum-based predictive panels for NOA and SO diagnosis. ROC curve analysis was conducted to distinguish NOA (*n* = 30) or SO (*n* = 30) from healthy controls (*n* = 30).

The predicted probability of developing NOA was calculated from the logit model using the TUG1 and miR-141 serum data with the equation Logit (P) = 4.2079–3.3906*TUG1–5.6528*miR-141. Using this equation, we constructed the ROC curve of the TUG1 + miR-141 combination (Fig. 7A). This panel distinguished NOA cases from fertile men with an AUC (95%CI) = 0.93 (0.845–1.012), *P* < 0.0001, a sensitivity of 90%, and a specificity of 96.67%. Interestingly, the panel has an excellent accuracy and outperforms the individual markers (which were only potential discriminators, AUCs < 0.9) in NOA diagnosis.

#### Construction of a predictive panel for SO

The predicted probability of developing SO was calculated from the logit model: Logit (P) = 1.9096–5.7169*TUG1 + 1.1936*miR-483. With this equation, the ROC curve (Fig. 7B) was constructed using the TUG1 and miR-483 serum levels. Interestingly, combining TUG1 + miR-483 distinguished SO cases from fertile men with an AUC (95%CI) = 0.972 (0.918–1.026), *P* < 0.0001. This panel performed with an optimal sensitivity of 100% and a specificity of 96.67%. Noticeably, this panel has an excellent accuracy and surpasses the individual markers in diagnosing SO.

### Studied serum markers are correlated with the clinical/laboratory data of NOA and SO patients

#### Correlation study in NOA patients

**Fig. 8:**
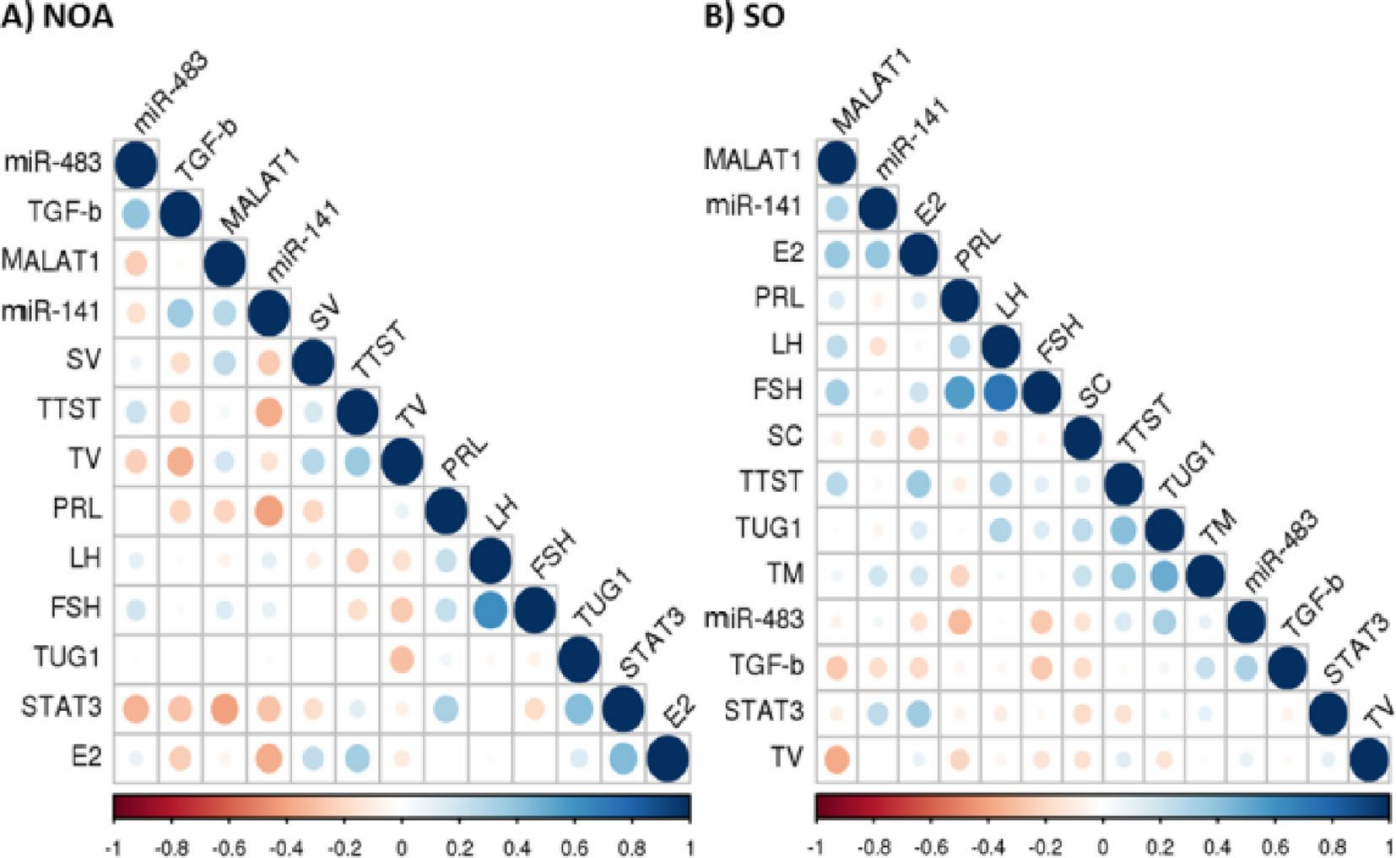
Correlation between serum markers and clinical, hormonal, and semen profiles in NOA and SO patients. **A** Correlation between parameters in the NOA group. **B** Correlation between parameters in the SO group. A correlation heatmap with a blue-red (cold-hot) scale. Correlations were computed using Spearman correlation. The heatmap was generated using https://www.sthda.com/english/. *E2* estradiol 2, *FSH* follicle-stimulating hormone, *LH* luteinizing hormone, *NOA* non-obstructive azoospermia, *PRL* prolactin, *SC* sperm count, *SO* severe oligozoospermia, *SV* semen volume, *TTST* total testosterone, *TM* total sperm motility, *TV* testicular volume.

Figure 8A represents a heatmap that demonstrates the correlations between parameters in the NOA group. As depicted in this figure, multiple substantial correlations were observed between the examined serum markers and the clinical and hormonal data in the NOA patients. Serum miR-483 expression was positively correlated with TGF-β1 protein levels (Spearman *r* = 0.39, *P* = 0.032). Serum STAT3 mRNA expression was correlated positively with TUG1 expression (*r* = 0.42, *P* = 0.019) and negatively with MALAT1 expression levels (*r* = − 0.41, *P* = 0.02). Interestingly, a negative correlation was recorded between serum TGF-β1 protein levels and testicular volume (*r* = − 0.36, *P* = 0.048). Such correlation was more reflected with the right and left testicular volume (*r* = − 0.43, *P* = 0.016; *r* = − 0.39, *P* = 0.035, respectively). Serum STAT3 expression was directly correlated with serum E2 (*r* = 0.43, *P* = 0.016). In contrast, negative correlations were noticed between miR-141 and TTST (*r* = − 0.375, *P* = 0.04), PRL (*r* = − 0.41, *P* = 0.023), and E2 levels (*r* = − 0.37, *P* = 0.044).

#### Correlation study in SO patients

Figure 8B portrays a heatmap representing the correlations between parameters in the SO group. Interestingly, we found a positive correlation between serum TUG1 and TTST levels (*r* = 0.41, *P* = 0.023). In addition, serum TUG1 was directly correlated with the total sperm motility (*r* = 0.49, *P* = 0.006) as well as progressive (*r* = 0.4, *P* = 0.026) and non-progressive (*r* = 0.42, *P* = 0.022) motilities. A negative correlation was recorded between serum MALAT1 and testicular volume (*r* = − 0.38, *P* = 0.036), which was particularly reflected with the left testicular volume (*r* = − 0.41, *P* = 0.023). In addition, serum MALAT1 and miR-141 were directly correlated with serum E2 levels (*r* = 0.37, *P* = 0.044; *r* = 0.38, *P* = 0.039, respectively). The Spearman rho coefficient values of all correlations are listed in Supplementary Tables S1 and S2.

## Discussion

Testicular reproductive illnesses do not yet have a viable treatment to stop or reverse their progression, and diagnosis is challenged by several limitations of the current diagnostic techniques^9–11^. Circulating lncRNAs and miRNAs have evolved as biomarkers and as phased and specific therapeutic targets of male infertility, empowered by their tissue-specific features, minimal invasiveness, and technical easiness and accessibility^47–50^. Given the limitations of semen and testicular markers^42^, recent research has focused on serum biomarkers in male infertility^16,51,52^; thus, we aimed to validate the serum expression signature of selected lncRNAs/miRNAs/target gene axes as less tedious, more accurate, clinically accessible, and cost-effective testing. At first, we examined the seminal plasma expression of markers as more reflective of the spermatogenesis status. Then we assessed these markers in serum. Intriguingly, we found that seminal plasma and serum expression levels of most of the tested markers were correlated among NOA and SO patients, suggesting their possible use as surrogate serum biomarkers in severe male infertility.

A notable finding of our study is the association of serum TUG1 and miR-141 with the risk of NOA and TUG1 and miR-483 with SO risk, highlighting them as potential diagnostic biomarkers. A combination-based prediction revealed that serum TUG1/miR-141 and TUG1/miR-483 appeared superior to individual markers in the diagnosis of NOA and SO, respectively. These serum-based panels performed with higher sensitivity (≥ 90%) and specificity (≥ 95%) in diagnosing NOA and SO and superseded other biomarkers such as cell free DNA or RNA, transcriptomics^53^, hormonal levels (LH, FSH, TTST, and PRL)^16^, and even serum miR-34a and NEAT1^16^. Our findings suggest that the proposed lncRNA/miRNA panel demonstrates strong diagnostic potential for NOA and SO; however, further validation is necessary before clinical implementation.

Our results recapitulate the altered expression of certain lncRNAs and miRNAs in testicular tissues, seminal plasma, or spermatozoa of male infertile patients^54–59^. A novel finding of our study is the observation of downregulated levels of TUG1 in the seminal plasma or sera of both SO and NOA patients compared to infertile men. Interestingly, low serum TUG1 levels were associated with the risk of NOA or SO among controls and correlated with TTST levels and reduced total, progressive, and non-progressive motility in SO patients. These results mirror previous findings that linked the knockdown of the Tug1 gene to impaired spermatogenesis in mice^23,24^. TUG–/– mice showed decreased sperm number and morphological sperm defects^23^. In addition, deletion of TUG1 impaired the tight junctions of SCs in high-fat diet mice^24^. Together, these results highlight the potential role of TUG1 in male infertility, warranting further investigation into its clinical utility.

In addition, our results showed MALAT1 as a potential differentially expressed gene (DEG) between NOA and SO men, with reciprocal levels of MALAT1 in both diseases, highlighting its disease-specific expression profile. Serum MALAT1 showed high discriminative ability between the two diseases, suggesting its possible usefulness in their differential diagnosis, with further validation needed. The low MALAT1 levels in SO patients are consistent with its low levels in semen samples from infertile men in another study^54^. Indeed, low seminal levels of HOTAIR and MALAT1 were associated with increased malondialdehyde and DNA damage in sperm^54^. Intriguingly, serum MALAT1 was correlated with testicular volume and serum E2 levels among enrolled SO patients. Similarly, MALAT1 in semen has been correlated with sperm quality parameters in infertile men^54^. Together, these results suggest the role of MALAT1 in the disease etiology. In contrast, our NOA patients showcased highly upregulated MALAT1 expression. This could be attributed to different regulatory pathways and disease microenvironments that led to the different MALAT1 expression patterns between the two diseases.

This study is the first to shed light on miR-483 as a serum biomarker of NOA and SO cases. We uncovered that serum miR-483-3p levels were augmented in both NOA and SO patients as shown in the seminal plasma. However, different association patterns of this miRNA were shown in the multivariate analysis. Only one study has provided evidence that miR-483-3p was differentially expressed in seminal plasma and testicular tissues of males with obstructive azoospermia^31^. On the other hand, the differential expression of miR-141 was previously studied in seminal plasma or sperm in infertile men compared to controls^55–59^; however, target gene analysis was not performed. In the current study, downregulated miR-141 was observed in the sera of both NOA and SO patients similar to their levels in the seminal plasma. Noticeably, serum miR-141 was associated with NOA risk and could be a potential biomarker. This result recapitulates that of a previous study where seminal plasma miR-141 levels showed diagnostic accuracy (AUC = 0.887) for NOA^56^. In contrast to our finding, a significant upregulation of miR-141 in semen plasma was detected in NOA patients^56,57^ and in patients with asthenozoospermia and oligoasthenozoospermia versus fertile controls, and have been correlated with abnormal sperm motility and quantity^58^. miR-141 was also elevated in seminal plasma and spermatozoa of NOA and SO patients and was negatively correlated with sperm concentration^59^. This discrepancy could be explained on the basis of different samples (serum vs. semen), sampling methods, analysis techniques (real-time PCR vs. microarray), normalizing controls, and potential confounding factors (e.g., age and lifestyle).

In this study, infertile men presented with a picture consistent with hypergonadotropic hypogonadism, as evidenced by elevated serum LH and FSH levels and reduced testicular size in NOA patients and increased serum FSH in SO patients relative to the fertile controls, reflecting impaired testicular function. However, TTST, E2, and PRL had comparable levels between fertile and infertile men. A notable finding of this study is the correlation between serum miR-141 and serum hormones (TTST, PRL, and E2) in NOA patients and E2 level in SO patients. These results link miR-141 to the endocrine disturbances of both diseases and could be useful in the clinical setting.

Altered miRNAs/target genes networks were among the critical mechanisms of impaired spermatogenesis^60–62^. Of particular interest, TGF-β1 is a multifactorial cytokine that has impeccable roles in fertility and the endocrine regulation of spermatogenesis^40,63^. TGF-β1 signaling occurs in the hub of miR-483 or miR-141 regulatory networks through multiple direct or indirect targets^34–36,64,65^. Indeed, genetic TGF-β1 deficiency deranges the hypothalamic-pituitary-gonadal axis function, suppressing LH synthesis, and subsequently reducing testosterone production in males^40,63^. While there is a controversy about the circulating TGF-β1 levels in infertile men^66–69^, we observed increased seminal plasma TGF-β1 only in NOA patients, while there were no significant alterations in serum TGF-β1 between infertile and fertile men. Discrepant TGF-β1 levels could be attributed to different sample types and processing, analysis methods, and confounding factors. Meanwhile, serum TGF-β1 was inversely correlated with testicular volume in the studied NOA patients. The noticeable positive correlation between serum miR-483 and TGF-β1 levels in NOA patients could be explained on the basis that dysregulation of miR-483 has been linked to disrupted TGF-β1 signaling through indirect targets^64,65^.

Another target of interest is STAT3. We found an elevated STAT3 expression in the seminal plasma or sera of both NOA and SO patients, with relatively higher serum levels in NOA than SO patients, suggesting STAT3 as a potential DEG. These results could be based on the fact that increased JAK2/STAT3 activity is coupled to the arrest of spermatogenesis, hampered expression of the SC junctions, and downregulation of antioxidant enzymes^41^. Interestingly, STAT3 is one of the novel targets of miR-141 as experimentally validated in cell lines^46,70^. The upregulation of STAT3 in NOA patients is consistent with MALAT1 upregulation and miR-141 downregulation, implying a possible role of the MALAT1/miR-141/STAT3 axis in the pathogenesis of NOA. On the other hand, STAT3 is also a target of miR-483, as evidenced in osteosarcoma cell lines^37^. The relatively lower levels of serum STAT3 in SO patients are consistent with the MALAT1 downregulation and miR-483 upregulation in these patients, implying a plausible role of the MALAT1/miR-483/STAT3 axis in the pathogenesis of SO. However, this hypothesis needs further investigation at the cellular level in testicular cell lines. In our study, the positive correlation of serum STAT3 with TUG1 and its negative correlation with MALAT1 among NOA patients suggest that STAT3 is subjected to other regulatory mechanisms.

Prior studies analyzing ncRNAs as potential biomarkers of male infertility^54–59^ had some methodological limitations, including potential selection bias in infertility clinics^54^, very small sample size^55^, less robust RNA quantification techniques (microarrays)^55^, genome-wide profiling, which might raise the risk of false positives^56^, tissue heterogeneity^55,56^, minimal and fragmented sperm RNA that is difficult to purely isolate^58^, and confounders like varicocele, infection, or medications that may not be fully excluded^59^. Our study has various advantages over earlier studies. First, we collected patient and control samples from two different hospitals to minimize potential selection bias, considered control group matching with both patient groups regarding age and smoking status, and applied rigorous exclusion criteria. Second, we utilized qPCR as a more robust analytical technique. Third, we analyzed both seminal plasma and serum markers and tested their correlations to establish more feasible serum-based markers.

Our study is a pioneer in demonstrating the signature of serum TUG1, MALAT1, miR-483, and miR-141, along with their targets TGF-β1 and STAT3, in SO and NOA patients, and their correlations with hormonal, clinical, and semen profiles. We also suggested novel serum-based predictive panels for assisting the diagnosis of both diseases. Additionally, we showed MALAT1 and STAT3 as potential DEGs for risk stratification. Given that ncRNA measurement is technically applicable in laboratory practice^71,72^, our findings could be clinically relevant in the precision medicine of male infertility and may have a potential value as supportive or confirmative to the standard diagnostic tests. This might afford promising tools for predicting sperm retrieval in men undergoing microsurgical testicular sperm extraction.

Nonetheless, we acknowledge several limitations in this study. First, we only carried out an in vivo investigation based on our bioinformatics analysis and previous evidence from in vitro studies. Second, despite the study’s adequate power, the sample size is relatively small due to the rigorous exclusion criteria, resulting in relatively small or large ORs. Third, we analyzed three groups without correcting the *P* value for multiple comparisons. Our study is considered preliminary research, and further large-scale studies are still warranted to verify our findings in independent cohorts. Longitudinal studies are also needed to assess the biomarker stability over time. Further in-depth elucidation of our investigated network using mechanistic studies in testicular cell lines could provide valuable information for understanding the molecular underpinnings of male infertility and expand its therapeutic repertoire.

Nevertheless, our results pinpoint a new strategy to empower the prediction, accurate diagnosis, and risk stratification of NOA and SO and provide serum-based assays that are more feasible for widespread clinical adoption and longitudinal monitoring during treatment or lifestyle interventions. The study findings also give insights into novel therapeutic approaches such as epigenetic targeted therapy.

## Conclusion

This study introduces a novel serum-based prediction panel of TUG1/miR-141 and TUG1/miR-483 that could be clinically tested to enhance the accuracy of NOA and SO diagnosis, respectively, warranting additional validation prior to clinical deployment. Serum MALAT1 and STAT3 could be used in stratifying NOA and SO cases, with further verification required. Serum miR-141 correlates with the hormonal profile in NOA patients, whereas serum TUG1 correlates with TTST levels and abnormal sperm motility in SO patients. Our results suggest a new horizon for helping empower the prediction and diagnosis of severe male infertility. This may also help predict the success of microsurgical testicular sperm retrieval and develop novel RNA-based personalized targeted treatments.

## Electronic supplementary material

Below is the link to the electronic supplementary material.

Supplementary Material 1

## Data Availability

All data generated or analyzed during this study are included in the manuscript and its supplementary files.
