# Supplementary material for: Serum expression signature of TUG1, MALAT1, miR-483, and miR-141 and their targets TGF-β1 and STAT3 in severe male factor infertility
Source: Sci Rep. 2025 May 27;15:18529. doi: 10.1038/s41598-025-03231-0 (PMC12116780; doi:10.1038/s41598-025-03231-0)
Supplement: Supplementary file 1 — Supplementary Material 1 [file 41598_2025_3231_MOESM1_ESM.docx]

**Supplementary Data**

**Supplementary Table S1. Correlations between serum markers and clinical parameters in NOA patients**

| Parameter | miR-483 | TGF-b1 | MALAT1 | miR-141 | SV | TTST | TV | PRL | LH | FSH | TUG1 | STAT3 | E2 |
| --- | --- | --- | --- | --- | --- | --- | --- | --- | --- | --- | --- | --- | --- |
| miR-483 | 1 |  |  |  |  |  |  |  |  |  |  |  |  |
| TGF-b1 | **0.39** | 1 |  |  |  |  |  |  |  |  |  |  |  |
| MALAT1 | -0.25 | -0.02 | 1 |  |  |  |  |  |  |  |  |  |  |
| miR-141 | -0.17 | 0.35 | 0.27 | 1 |  |  |  |  |  |  |  |  |  |
| SV | 0.06 | -0.18 | 0.25 | -0.26 | 1 |  |  |  |  |  |  |  |  |
| TTST | 0.21 | -0.22 | 0.04 | **-0.37** | 0.17 | 1 |  |  |  |  |  |  |  |
| TV | -0.24 | **-0.36** | 0.18 | -0.15 | 0.27 | **0.36** | 1 |  |  |  |  |  |  |
| PRL | -0.01 | -0.22 | -0.22 | **-0.41** | -0.21 | 0 | 0.08 | 1 |  |  |  |  |  |
| LH | 0.1 | 0.01 | -0.07 | 0.1 | -0.1 | -0.23 | -0.16 | 0.22 | 1 |  |  |  |  |
| FSH | 0.19 | 0.02 | 0.14 | 0.09 | -0.01 | -0.18 | -0.26 | 0.23 | 0.62 | 1 |  |  |  |
| TUG1 | -0.02 | 0.01 | 0 | 0.02 | -0.01 | -0.01 | -0.3 | 0.05 | -0.04 | -0.07 | 1 |  |  |
| STAT3 | **-0.35** | -0.29 | **-0.41** | -0.29 | -0.18 | 0.12 | -0.08 | 0.32 | -0.01 | -0.19 | **0.42** | 1 |  |
| E2 | 0.08 | -0.25 | -0.06 | **-0.37** | 0.24 | 0.33 | -0.11 | 0 | -0.02 | -0.01 | 0.15 | **0.43** | 1 |

Correlation was conducted using Spearman correlation. Spearman rho coefficient (r) values are presented. Bold means statistical significance (*P* < 0.05). E2, estradiol 2; FSH, follicle-stimulating hormone; LH, luteinizing hormone; PRL, prolactin; SV; semen volume; TTST, total testosterone; TV, testicular volume.

**Supplementary Table S2 Correlations between serum markers and clinical parameters in SO patients**

| Parameter | MALAT1 | miR-141 | E2 | PRL | LH | FSH | SC | TTST | TUG1 | TM | miR-483 | TGF-b1 | STAT3 | TV |
| --- | --- | --- | --- | --- | --- | --- | --- | --- | --- | --- | --- | --- | --- | --- |
| MALAT1 | 1 |  |  |  |  |  |  |  |  |  |  |  |  |  |
| miR-141 | 0.3 | 1 |  |  |  |  |  |  |  |  |  |  |  |  |
| E2 | **0.37** | **0.38** | 1 |  |  |  |  |  |  |  |  |  |  |  |
| PRL | 0.14 | -0.07 | 0.12 | 1 |  |  |  |  |  |  |  |  |  |  |
| LH | 0.25 | -0.16 | 0.03 | 0.26 | 1 |  |  |  |  |  |  |  |  |  |
| FSH | 0.33 | 0.03 | 0.2 | **0.55** | **0.72** | 1 |  |  |  |  |  |  |  |  |
| SC | -0.07 | -0.13 | -0.25 | -0.05 | -0.12 | -0.05 | 1 |  |  |  |  |  |  |  |
| TTST | 0.27 | 0.03 | 0.36 | -0.09 | 0.27 | 0.11 | 0.13 | 1 |  |  |  |  |  |  |
| TUG1 | 0.01 | -0.06 | 0.15 | -0.02 | 0.29 | 0.14 | 0.25 | **0.41** | 1 |  |  |  |  |  |
| TM | 0.06 | 0.19 | 0.18 | -0.22 | 0.03 | -0.02 | 0.21 | **0.36** | **0.49** | 1 |  |  |  |  |
| miR-483 | -0.07 | 0.05 | -0.16 | -0.32 | -0.02 | -0.27 | -0.15 | 0.16 | 0.32 | 0.09 | 1 |  |  |  |
| TGF-b | -0.27 | -0.18 | -0.2 | -0.05 | -0.04 | -0.27 | -0.19 | -0.03 | 0.05 | 0.22 | 0.31 | 1 |  |  |
| STAT3 | -0.09 | 0.25 | 0.35 | -0.07 | -0.1 | -0.04 | -0.19 | -0.16 | 0.04 | 0.1 | 0.01 | -0.07 | 1 |  |
| TV | **-0.38** | -0.01 | 0.08 | -0.22 | -0.05 | -0.15 | -0.16 | 0.12 | -0.15 | -0.02 | 0.09 | -0.04 | 0.1 | 1 |

Correlation was conducted using Spearman correlation. Spearman rho coefficient (r) values are presented. Bold means significant difference (*P* < 0.05). E2, estradiol 2; FSH, follicle-stimulating hormone; LH, luteinizing hormone; PRL, prolactin; SC, sperm count; TTST, total testosterone; TM, total sperm motility; TV, testicular volume.
